# Olig3 Is Not Involved in the Ventral Patterning of Spinal Cord

**DOI:** 10.1371/journal.pone.0111076

**Published:** 2014-10-28

**Authors:** Zijing Liu, Xuemei Hu, Chengcheng Huang, Kang Zheng, Hirohide Takebayashi, Cheng Cao, Mengsheng Qiu

**Affiliations:** 1 Department of Anatomical Sciences and Neurobiology, University of Louisville, Louisville, KY, United States of America; 2 Beijing Institute of Biotechnology, Beijing, PR China; 3 Institute of Developmental and Regenerative Biology, Zhejiang Key Laboratory of Organ Development and Regeneration, Hangzhou Normal University, Hangzhou, PR China; 4 Division of Neurobiology and Anatomy, Graduate School of Medical and Dental Sciences, Niigata University, Niigata, Japan; Université Pierre et Marie Curie-Paris6, INSERM, CNRS, France

## Abstract

At embryonic stages, Olig3 is initially expressed in the dorsal-most region of the spinal cord, but later in the ventral marginal zone as well. Previous studies indicated that Olig3 controlled the patterning of dorsal spinal cord and loss of Olig3 function led to the re-specification of dI2 and dI3 neurons into dI4 interneurons. However, the role of Olig3 in regulating the development of ventral spinal cord has remained unknown. BrdU labeling demonstrated that ventral Olig3 was expressed in the post-mitotic neurons and Olig3+ cells seen at late embryonic stages were born at the earlier stage but remained in the marginal zone throughout embryogenesis. Loss-of-function and gain-of-function experiment indicated that Nkx2.2 regulated the expression of Olig3 in V3 interneurons. However, *Olig3* mutation didn’t apparently affect the generation and migration of ventral neurons. These findings suggest that Olig3 plays different roles in regulating the development of dorsal and ventral spinal cord.

## Introduction

In the past few years, considerable progress has been made in understanding the patterning of the spinal cord ventricular zone. Based on the expression of distinct transcription factors, the dorsal neuroepithelium is divided into six progenitor domains (dp1–6), with each domain producing a specific dorsal interneuron phenotype (dI1–6) which can be recognized by a unique combination of expression of neural progenitor markers [1], [2], [3]. The ventral ventricular zone can be divided into five distinct domains (p0–p3 and pMN), each of which generates a unique neuronal type [4], [5].

*Olig* genes are a sub-family of the bHLH transcription factors that consist of *Olig1–3*. Olig1 and Olig2 are selectively expressed in pMN domain and play key roles in controlling the specification of both motor neurons and oligodendrocytes in the spinal cord. Loss of Olig2 in mutant completely inhibited the generation of motor neurons and oligodendrocyte, whereas *Olig1* knockout mice showed the delay of oligodendrocyte differentiation [6], [7], [8]. *Olig3* was identified as the third member of *Olig* family besides *Olig1* and *Olig2*. Alignment of bHLH domains in *Olig* genes showed that the bHLH domain of *Olig3* is highly related to *Olig2* in structure [9], but the expression pattern of Olig3 is complementary to that of Olig1 and Olig2 [10]. Previous studies showed that Olig3 was involved in the patterning of dorsal spinal cord and hindbrain [11], [12], [13]. Loss of Olig3 function would impair the development of class A (dI1–dI3) neurons and mis-specify dI2–dI3 neurons into dI4 interneurons. In *Olig3* mutant hindbrain, not only the generation of inferior olive nucleus, nucleus of the solitary tract and brainstem (nor)adrenergic centers is completely inhibited, but the formation of four mossy-fiber nuclei is reduced [11].

Besides dorsal Olig3 expression, Olig3 protein was also detected in ventral spinal cord [10], but its function in the development of ventral spinal cord has remained unknown. In this study, we first examined the expression pattern of Olig3 in embryonic chick and mouse spinal cord in details. BrdU labeling demonstrated that ventral Olig3 was expressed in postmitotic neurons at the early embryonic stage, and these Olig3+ neurons remained in the marginal zone throughout embryogenesis. Both loss- and gain-of-function experiments indicated that Nkx2.2 regulated the expression of Olig3 in V3 interneurons. However, *Olig3* mutation didn’t apparently change the generation and migration of ventral neurons. These findings suggest that the development of dorsal and ventral neurons has different requirement for Olig3.

## Materials and Methods

### Animals

The *Nkx2.2* or *Olig3* homozygous null embryos were obtained by the interbreeding of double heterozygous animals. The noon on the day of vaginal plug discovery was designated as embryonic day 0.5 (E0.5). The *Nkx2.2* targeted mutant transgenic mouse line was obtained from Dr. John Rubenstein (University of California, San Francisco, CA). Genomic DNA extracted from embryonic tissues or mouse tails was used for genotyping by Southern analysis (Figure S1). Genomic DNA of *Nkx2.2* mutant was digested by ApaI, genomic DNA of *Olig3* mutant was digested by HindIII and SpeI. Genotyping of *Olig3* and *Nkx2.2* loci was described earlier [11], [14]. All animal use was approved by the Institutional Animal Care and Use Committee at the University of Louisville (IACUC: 12034).

### *In situ* RNA hybridization

Spinal cord tissues at the thoracic level were isolated from mouse or chick embryos and then fixed in 4% paraformaldehyde at 4°C overnight. Following fixation, tissues were transferred to 20% sucrose in PBS overnight, embedded in OCT media and then sectioned (20 µm thickness) on a cryostat. Adjacent sections from the wild-type and mutant embryos were subsequently subjected to *in situ* hybridization (ISH). ISH was performed as described in [11]. Chick Sim1 antisense probe was made from *Sim1* cDNA plasmid with T3 RNA polymerase (sequence +440–+960). The sense control probe was generated with T7 RNA polymerase. No signal was detected by sense control probe.

### Immunofluorescent staining

Spinal cord tissues at the thoracic level were isolated from mouse or chick embryos and then fixed in 4% paraformaldehyde at 4°C overnight. Following fixation, tissues were transferred to 20% sucrose in PBS overnight, embedded in OCT media and then sectioned (20 µm thickness) on a cryostat. After rinsing with PBS, sections were permeabilized in 0.1% Triton X-100 in PBS (omit this step if the antigen is a membrane lipid) for 10 minutes, rinsed out the excessive Triton with PBS, incubated in blocking solution (5% normal serum in PBS plus 1% BSA) at room temperature for 1 hour, and incubated in diluted primary antibody in blocking solution at 4°C overnight. Next day, sections were washed in PBS for three times 10 minutes each, and incubated in the secondary antibody in blocking solution at room temperature for 1 hour. After incubation, sections were washed in PBS for three times 10 minutes each, before they were mounted in Mowiol mounting medium on glass slides. The staining was examined under a Nikon fluorescence microscope.

Anti-Nkx2.2 (1∶40, 74.5A5), anti-BrdU (1∶40, G3G4), anti-Islet1 (1∶100, 40.2D6) monoclonal antibodies or hybridoma cells were obtained from the Developmental Studies Hybridoma Bank. Rabbit polyclonal antibody anti-Olig2 (1∶20000) was generously provided by Drs. Charles Stiles and David Rowitch (Harvard Medical School, Boston, MA and University of California San Francisco, San Francisco, California). Rat polyclonal antibody anti-Olig3 (1∶2000) was described in [10]. Guinea-pig polyclonal antibody anti-Chx10 (1∶5000) was generously provided by Dr. Pfaff Samuel (The Salk Institute for Biological Studies, La Jolla, CA). The Alexa-488 or Alexa-594 conjugated secondary antibodies were obtained from Molecular Probes (Eugene, OR).

### BrdU labeling

For BrdU labeling experiments, pregnant mice were injected intraperitoneally with 75 µg BrdU (Sigma) per gram body weight. Slides were first immunostained with anti-Olig3 and Alexa-594 conjugated secondary antibody as described above. Slides were then washed 3×10 min with PBS and fixed in absolute methanol for 10 min at RT. The air-dried slides were treated with 2 N HCl for 10 min at RT, washed 2×5 min with PBS, and neutralized in 0.1 M sodium borate buffer (pH 8.0) for 10 min at RT. Following a brief wash with PBS, sections were labeled with anti-BrdU supernatant and Alexa-488 conjugated secondary antibody. Data shown are representative of independent experiments performed in triplicate on three animals per time point.

### In Ovo electroporation

Fertilized White Leghorn chicken eggs were obtained from Charles River and incubated at 38°C in a humidified incubator. Embryos were staged according to the criteria set by Hamburger and Hamilton [15].

Full-lengths mouse *Nkx2.2* cDNA was subcloned into the replication-competent retroviral vector RCASBP (B). For *in ovo* electroporation, about 1.5 µl (1 µg/µl) of expression vectors was injected into stage 11–13 chicken embryos with the aid of Picospritzer III instrument. The injected embryos were then subjected to three short-pulses of electrical shock (25 V, 50 msec for each pulse) and allowed to develop for various time periods before they were fixed in 4% PFA for gene expression studies.

### Cell counting and statistical analyses

At least three distinct animals were used to prepare spinal cord sections for each genotype. Only cells containing nuclei and showing levels of staining clearly above background were counted. Values were presented as mean ± SD. The differences in values were considered to be significant at p<0.05 by Student’s *t* test.

## Results

### Olig3 expression pattern in embryonic spinal cord

**Figure 1:**
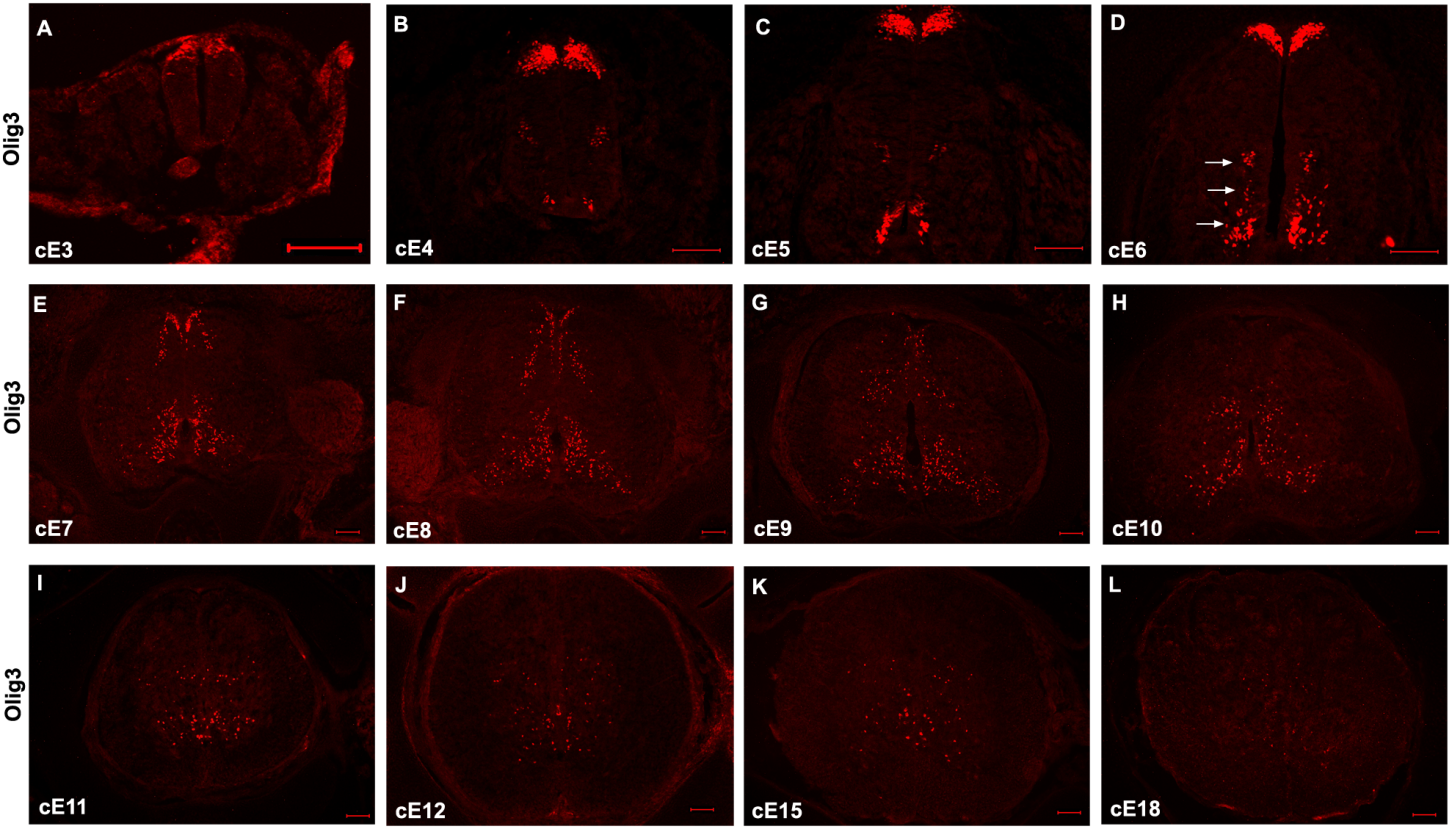
Developmental expression of Olig3 in the embryonic chick spinal cords at the thoracic level. Cross sections from various embryonic chick spinal cord tissues were subjected to immunofluorescent staining with rat anti-Olig3 antibody. (A) cE3. (B) cE4. (C) cE5. (D) cE6. (E) cE7. (F) cE8. (F) cE9. (G) cE10. (H) cE11. (I) cE12. (J) cE15. (K) cE18. The dorsal part is up. Bars, 100 µm.

**Figure 2:**
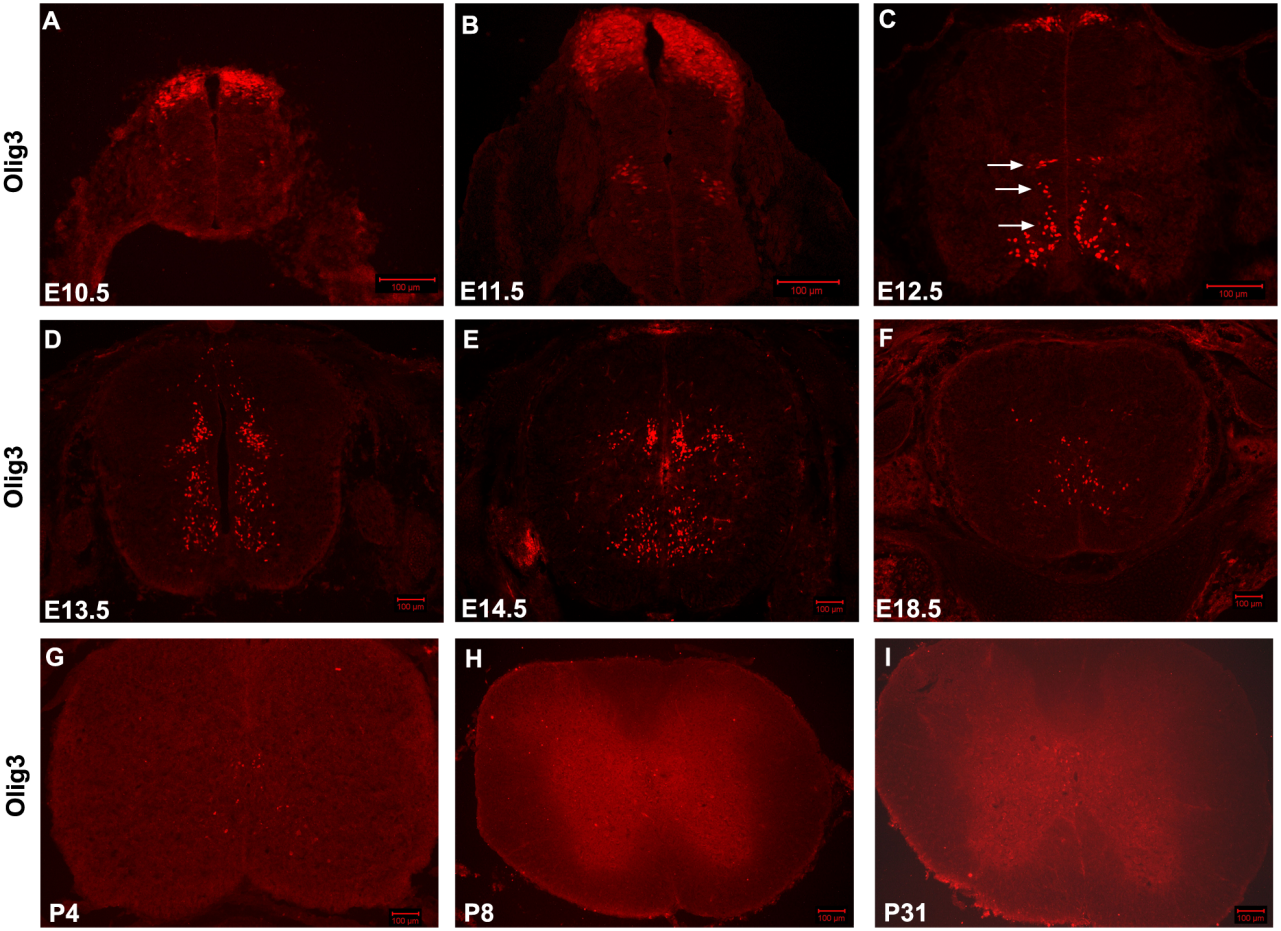
Olig3 expression pattern in the developing mouse spinal cord. Cross sections from various embryonic mouse spinal cord tissues were subjected to immunofluorescent staining with rat anti-Olig3 antibody. (A) E10.5. (B) E11.5. (C) E12.5. (D) E13.5. (E) E14.5. (F) E18.5. (G) P4. (H) P8. (I) P31. Olig3 was initially expressed in the dorsal-most neuroepithelial cells at E10.5, and transiently expressed in three ventral groups at E12.5 (Shown by arrows). At E13.5, Olig3 was expressed in the lateral marginal zone of the entire spinal cord along dorsal-ventral axis. The expression pattern of Olig3 in the spinal cord is maintained from E14.5 to E18.5. Olig3 expression remained detectable at postnatal day 4 (P4) but not at P8 and later stages. The dorsal part is up. Bars, 100 µm.

As a first step to elucidate the function of Olig3 in spinal neurogenesis, we carried out detailed analysis on Olig3 expression in embryonic chick spinal cord at various development stages. Olig3 was initially expressed in the dorsal-most neuroepithelial cells at cE3 (Figure 1A). Starting at cE4, Olig3 expression was also detected in the marginal zone at the intermediate and ventral-most regions of spinal cord, whereas dorsal Olig3 expression was observed in a broad region (Figure 1B). At cE5, some Olig3+ cells were also transiently detected in the ventral ventricular zone (Figure 1C). Three distinct clusters of Olig3+ neurons appeared at the lateral margin derived from p0, p2 and p3 domain at cE6 (arrows in Figure 1D, S2). By cE7, dorsal Olig3+ cells were distributed along the dorsal ventricular zone and lateral mantle zone, whereas the ventral Olig3+ cells spread to adjacent mantle zone (Figure 1E). At cE9, some Olig3+ cells have distributed into the ventral-lateral border of the spinal cord (Figure 1G). At cE10, dorsal-most Olig3 expression was completely lost, and Olig3 was only expressed in the base of dorsal horn and ventral gray matter (Figure 1H). This expression pattern was maintained at later stages, although its expression level gradually declined (Figure 1I–L). By cE18, only weak Olig3 expression could be detected (Figure 1L). Similar Olig3 spatiotemporal expression pattern was also found in embryonic mouse spinal cord (Figure 2).

To test our hypothesis that Olig3 is expressed in interneurons, we first performed double immunostaining experiments on embryonic spinal cord sections with anti-Olig3 and NeuN, a pan-neuronal marker which labels both mature and immature neurons. Consistent with our hypothesis, 92% Olig3+ cells co-expressed NeuN in cE9 spinal cords (Figure S3A–C). To examine the possibility that Olig3 might also be expressed in glial cells, we performed double immunostainging on embryonic spinal cord with anit-Olig3 and anti-Olig2 (oligodendrocyte-specific marker). Our studies demonstrated that Olig3*+* cells were Olig2-negative at cE12 (Figure S3D–F).

### Proliferation and birth-dating analyses of Olig3+ neurons in the ventral spinal cord

**Figure 3:**
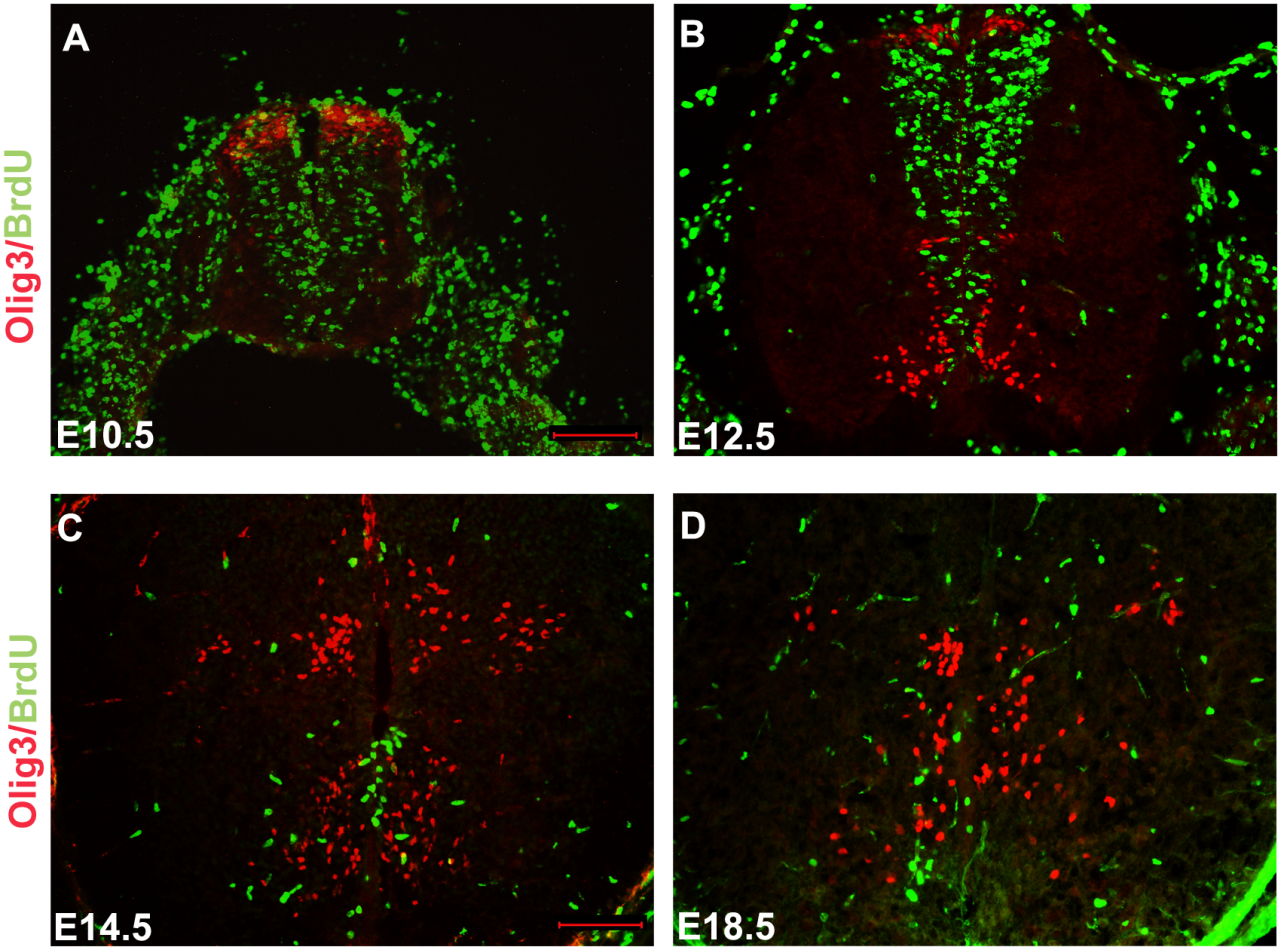
Short-term BrdU birth-dating analysis of Olig3+ cells in the developing mouse spinal cords. Mouse embryos from E10.5 (A), E12.5 (B), E14.5 (C), and E18.5 (D) were pulse-labeled with BrdU for 2 hours. Transverse spinal cord sections were subjected to double immunolabeling with anti-BrdU (green) and anti-Olig3 (red). Many Olig3/BrdU double-positive cells were observed at the dorsal-most domain of spinal cord at E10.5. In contrast, no Olig3/BrdU double-positive cells could be found at E12.5, E14.5 and E18.5. The dorsal part is up. Bars, 100 µm.

Based on the spatiotemporal patterns of Olig3 expression, we hypothesized that Olig3+ cells in the ventral marginal zone represent postmitotic immature neurons. To test this hypothesis, we performed short pulse of BrdU labeling experiment. In this experiment, female mice pregnant with E10.5, E12.5, E14.5 and E18.5 embryos were injected intraperitoneally with BrdU for 2 hours before embryos were processed. The 2-hour labeling is long enough to allow the incorporation of BrdU into the DNA of dividing cells but too brief to allow the departure of the proliferating cells from the ventricular zone (it takes more than two hours for neuroepithelial cells to progress from S-phase to the completion of M-phase). No Olig3/BrdU double-positive cells could be found in the spinal cord at E12.5, E14.5 and E18.5 (Figure 3B–D), suggesting Olig3+ cells in the ventral marginal zone are postmitotic neurons. There were Olig3*+*/BrdU+ cells observed at dorsal-most domain of spinal cord at E10.5 (Figure 3A), indicating that dorsal Olig3*+* cells are proliferating progenitor cells at early stage.

**Figure 4:**
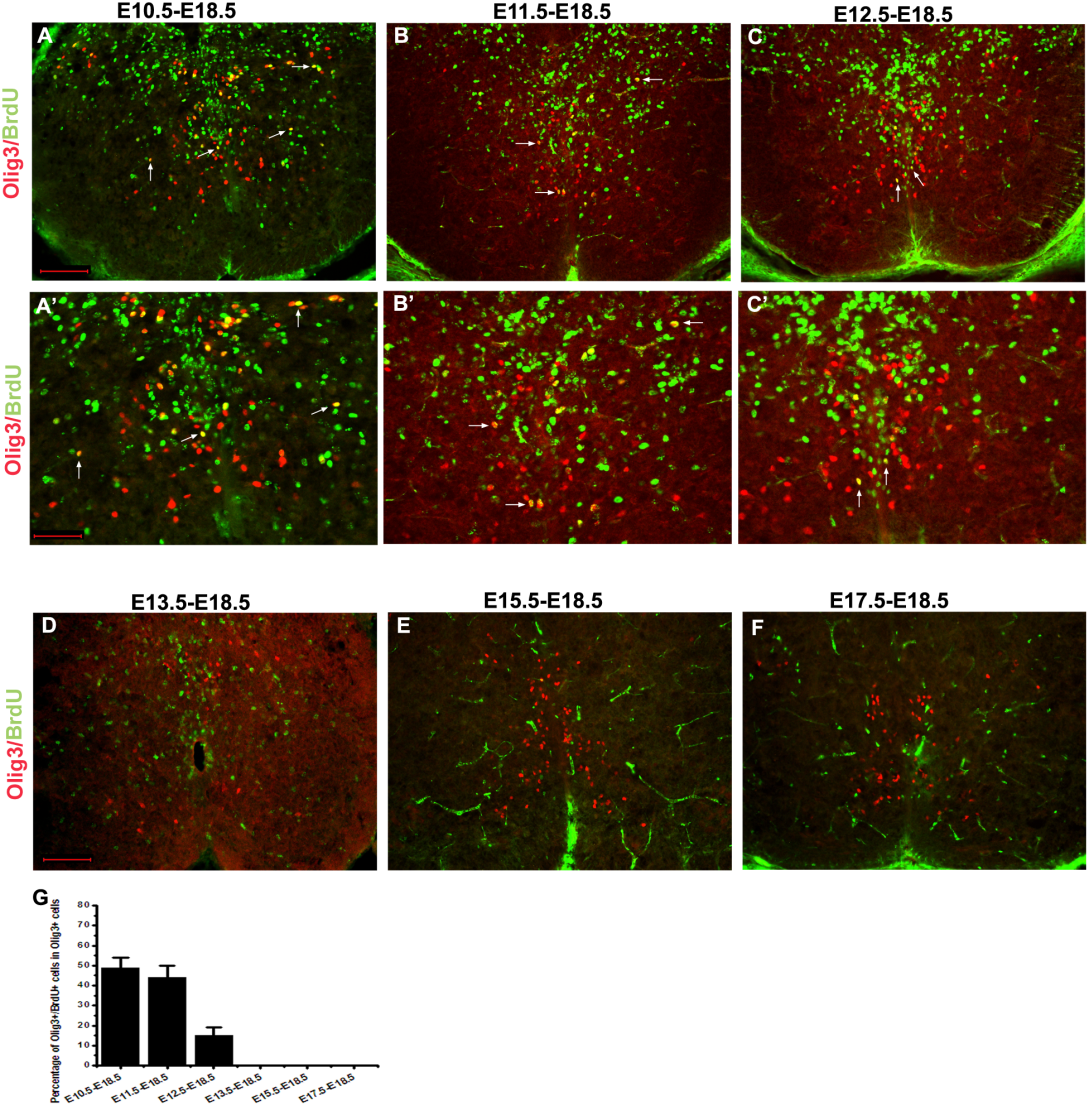
Long-term BrdU birth-dating analysis of Olig3+ cells in the developing mouse spinal cords. BrdU was injected into pregnant mice at various embryonic stages and embryos were harvested at E18.5. Transverse spinal cord sections were subjected to double immunolabeling with anti-BrdU (green) and anti-Olig3 (red). Spinal cord section from mice injected at E10.5, E11.5 and E12.5 contained BrdU+/Olig3+ cells. However, injection after E13.5 did not produce any Olig3/BrdU double-positive cells. A’–C’ are the high-magnification images of A–C, respectively. Double positive cells are indicated with arrows. (G) The percentage of Olig3+ cells that co-express BrdU was calculated. The dorsal part is up. Bars: A–F are 100 µm; A’–C’ are 50 µm.

It would be interesting and important to determine whether Olig3+ cells at the marginal zone seen at late embryonic stages (e.g. E18.5) were newly generated from local ventricular zone, or were generated at early stages but remained throughout embryogenesis. To distinguish between these two possibilities, we performed long-term BrdU birthdating analysis of Olig3+ cells. BrdU was injected into pregnant mice at different stages, and embryos were harvested at E18.5. BrdU+/Olig3+ cells were found in the intermediate and ventral regions of spinal cord when BrdU was injected at E10.5 or E11.5, but only detected in the ventral region with E12.5 injection (Figure 4A–C). Injection after E13.5 yielded no Olig3*+*/BrdU+ cells (Figure 4D–F). Therefore, Olig3+ cells seen at late embryonic stages were born from E10.5 to E12.5, with the majority born being born at E10.5 and E11.5. The Olig3+ neurons remained in the marginal zone throughout embryogenesis, suggesting that Olig3 may regulate the differentiation of these positive cells located at the mantle zone.

### The majority of ventral Olig3*+* cells are V3 interneurons

**Figure 5:**
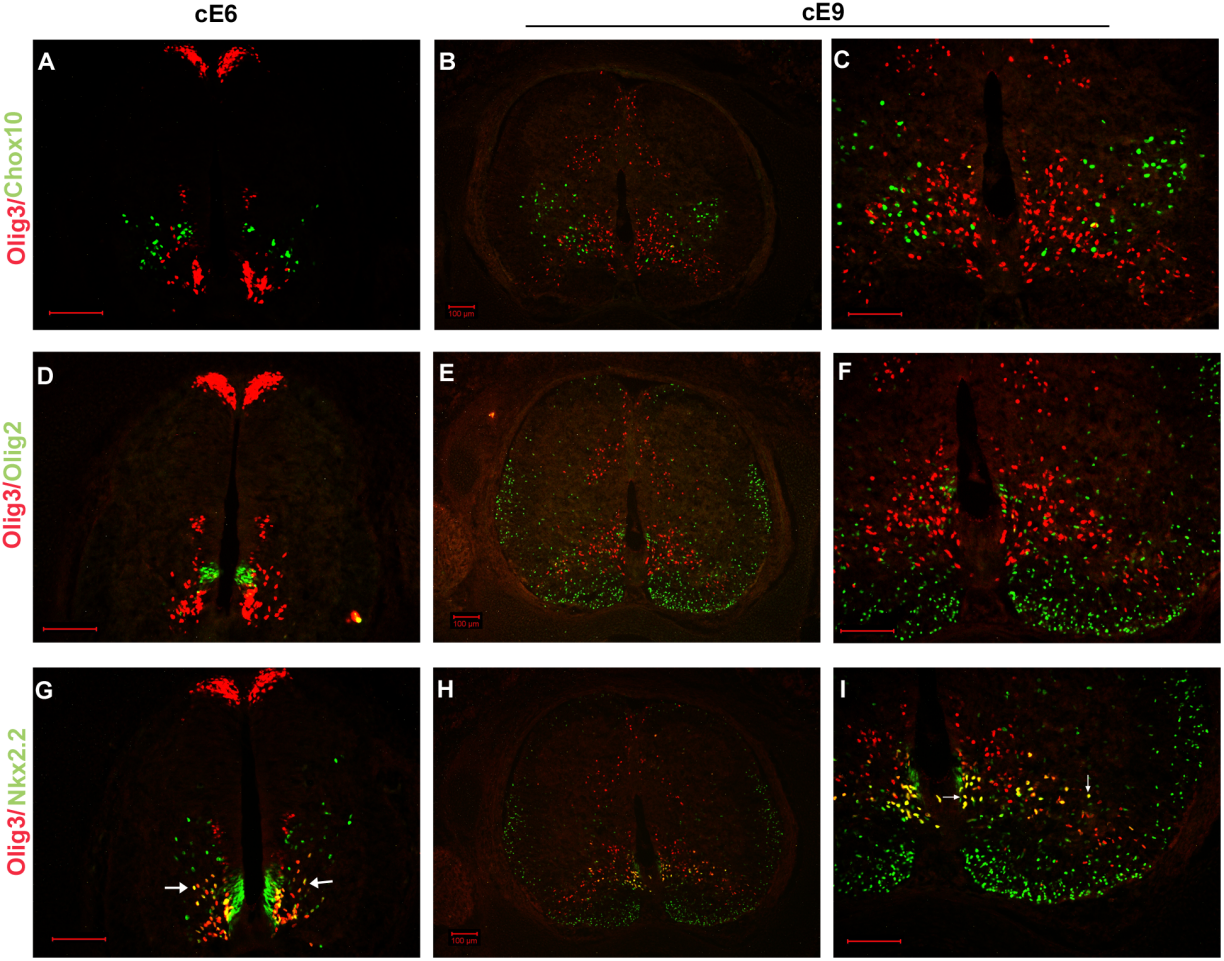
Olig3 is expressed in the cells derived from p2 and p3 domains. cE6 and cE9 chicken spinal cord sections were double-immunostained with anti-Olig3 (red), anti-Chx10 (green), anti-Olig2 (green) and anti-Nkx2.2 (green). Olig3 was expressed in the cells derived from p2 and p3 domains of ventricular zone, but not in *Olig2*+ motor neuron progenitor cells and oligodendrocytes. **C, F, I** are the high power views of **B, E, H,** respectively, in the ventrolateral region. Double positive cells are indicated with arrows. The dorsal part is up. Bars, 100 µm.

To define the Olig3 expression domain in the developing ventral spinal cord, we performed double labeling experiments to compare Olig3 expression with different region marker, such as Chx10 (V2 Interneuron), Olig2 (Motor Neuron) and Nkx2.2 (V3 Interneuron). At cE6, small subsets of Olig3+ cells were located at lateral margin of p2 domain but did not co-express Chx10 (Figure 5A). At cE9, Olig3 protein was not detected in radial migrating Chx10*+* V2 interneurons (Figure 5B, C). Our results showed that Olig3 and Olig2 were not expressed in the same population of cells in the spinal cord (Figure 5D–F). Since Olig2+ cells represent motor neuron progenitor cells in the ventricular zone and oligodendrocyte cells outside the ventricular zone [16], [17], , it is unlikely that Olig3 is expressed in these two cell types, further supporting the idea that Olig3 is specifically expressed in interneurons.

At cE6, a large number of Olig3+ cells co-expressed Nkx2.2 outside the p3 progenitor domain (Figure 5G), and these Olig3+/Nkx2.2+ cells were confined to the gray matter even at cE9 (Figure 5H, I), suggesting that these double positive cells are V3 interneurons. There was no double positive cell detected in the white matter, suggesting that Olig3+ cells are not oligodendrocytes [19]. According to their position and intrinsic properties, V3 interneurons can be divided into ventral and dorsal subpopulations [20], [21]. Double-labeling experiment showed that Olig3+/Nkx2.2+ cells were primarily located in lamina VIII and belonged to the ventral V3 interneurons. Based on these data, we found that in the ventral spinal cord, Olig3 was expressed in the cells derived from p2 and p3 domain of ventricular zone, in which p3 domain was the major site of origin.

### Olig3 functions downstream of Nkx2.2 in V3 interneurons

**Figure 6:**
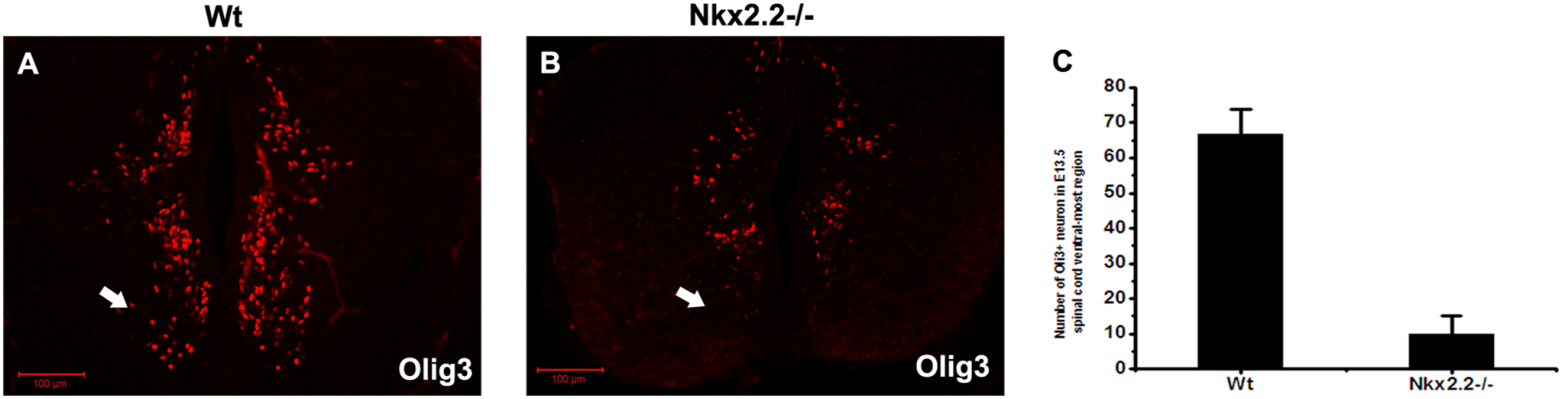
Nkx2.2 controls Olig3 expression in V3 interneurons. Transverse spinal cord sections from wild-type (A) and *Nkx2.2*−*/*− (B) embryos were subjected to immunolabeling with anti-Olig3 antibody. In E13.5 *Nkx2.2* knockout mice, Olig3 expression was almost completely lost in ventral-most region of spinal cord. Arrows indicate the region in which Olig3 expression is affected. (C) The number of Olig3+ cells in the ventral-most region of E13.5 wild type and *Nkx2.2*−*/*− spinal cords. p<0.001. The dorsal part is up. Bars, 100 µm.

Previous study reported that dorsal Olig3*+* cells could migrate to ventral-most region of the spinal cord, adjacent to p3 domain [22]. To determine whether ventral Olig3*+* neurons were generated from local regions or emigrated from dorsal spinal cord, we examined the expression of Olig3 in *Nkx2.2* mutants in which the entire p3 domain was transformed into pMN domain and V3 interneurons were mis-specified into motor neurons [23], [24]. Consistently, the loss of V3 interneurons was associated with the absence of ventral-most Olig3 expression in E13.5 *Nkx2.2*−*/*− spinal cord (Figure 6), suggesting that Nkx2.2 controls Olig3 expression in the ventral-most cells.

**Figure 7:**
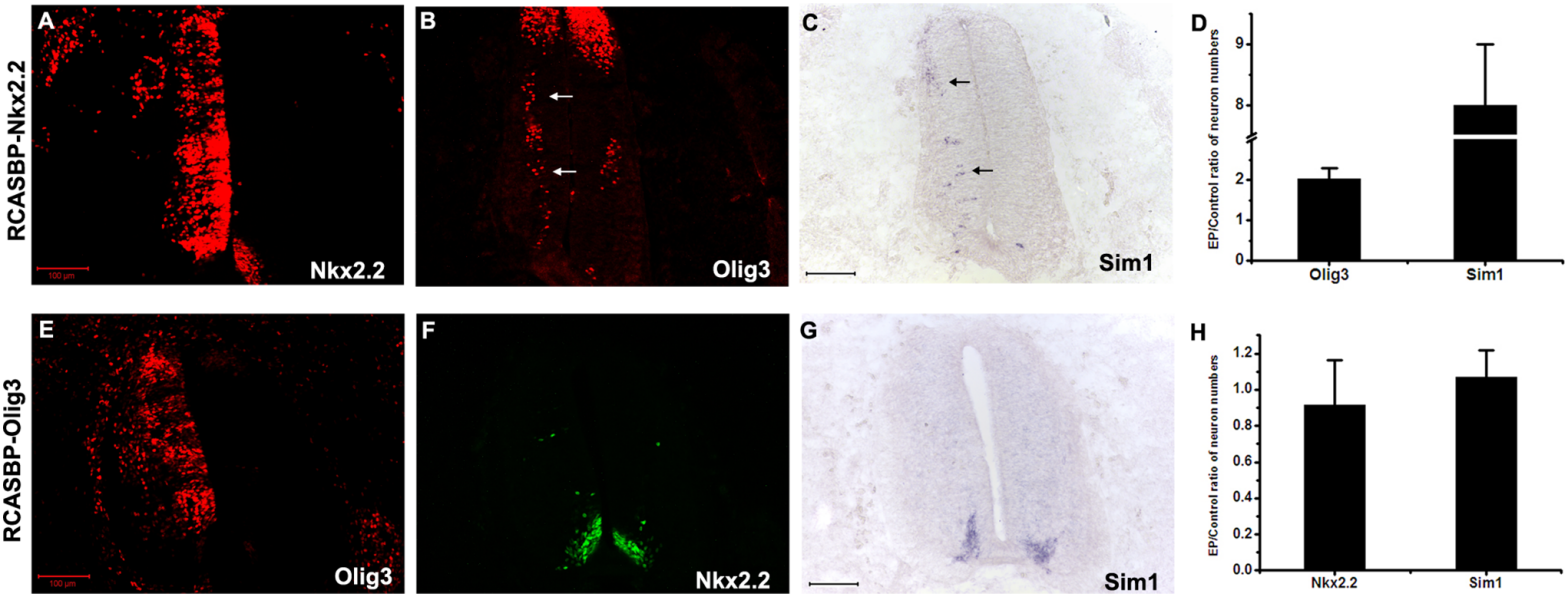
Nkx2.2 overexpression induces ectopic Olig3 expression. (A–C) The spinal cords of cE2 chick embryos were electroporated in ovo with replication competent RCASBP retroviral vectors harboring *Nkx2.2* gene. The embryos were harvested 2 days later (cE4). Over-expression of Nkx2.2 induced ectopic expression of Olig3 and V3 interneuron marker Sim1. Ectopic Olig3+ and Sim1+ neurons are indicated with arrows. (D) The ratio of the positive cells generated in the Nkx2.2 electroporated (EP) and the control side of the spinal cord. p<0.005. (E–G) Over-expression of Olig3 did not affect Nkx2.2 and Sim1 expression. (H) The ratio of neuron numbers generated on Olig3 electroporated side and the control side. p>0.05. The dorsal part is up. Bars, 100 µm.

To confirm this possibility, cE2 chick spinal cords were electroporated with *Nkx2.2* expression plasmid *in ovo*. Two days after electroporation (cE4), embryos were harvested and analyzed. Consistent with the notion that *Olig3* is a downstream gene of *Nkx2.2* during the development of V3 interneurons, over-expression of Nkx2.2 significantly induced the expression of Olig3 and increased the number of Sim1+ V3 interneurons at cE4 (Figure 7A–D). In contrast, over-expression of Olig3 was not able to affect the number and migration of Nkx2.2+ and Sim1+ V3 interneurons (Figure 7E–H), supporting the idea that *Olig3* was the downstream target of *Nkx2.2* in p3 domain.

### *Olig3* mutation has no apparent effect on the development of ventral neurons

**Figure 8:**
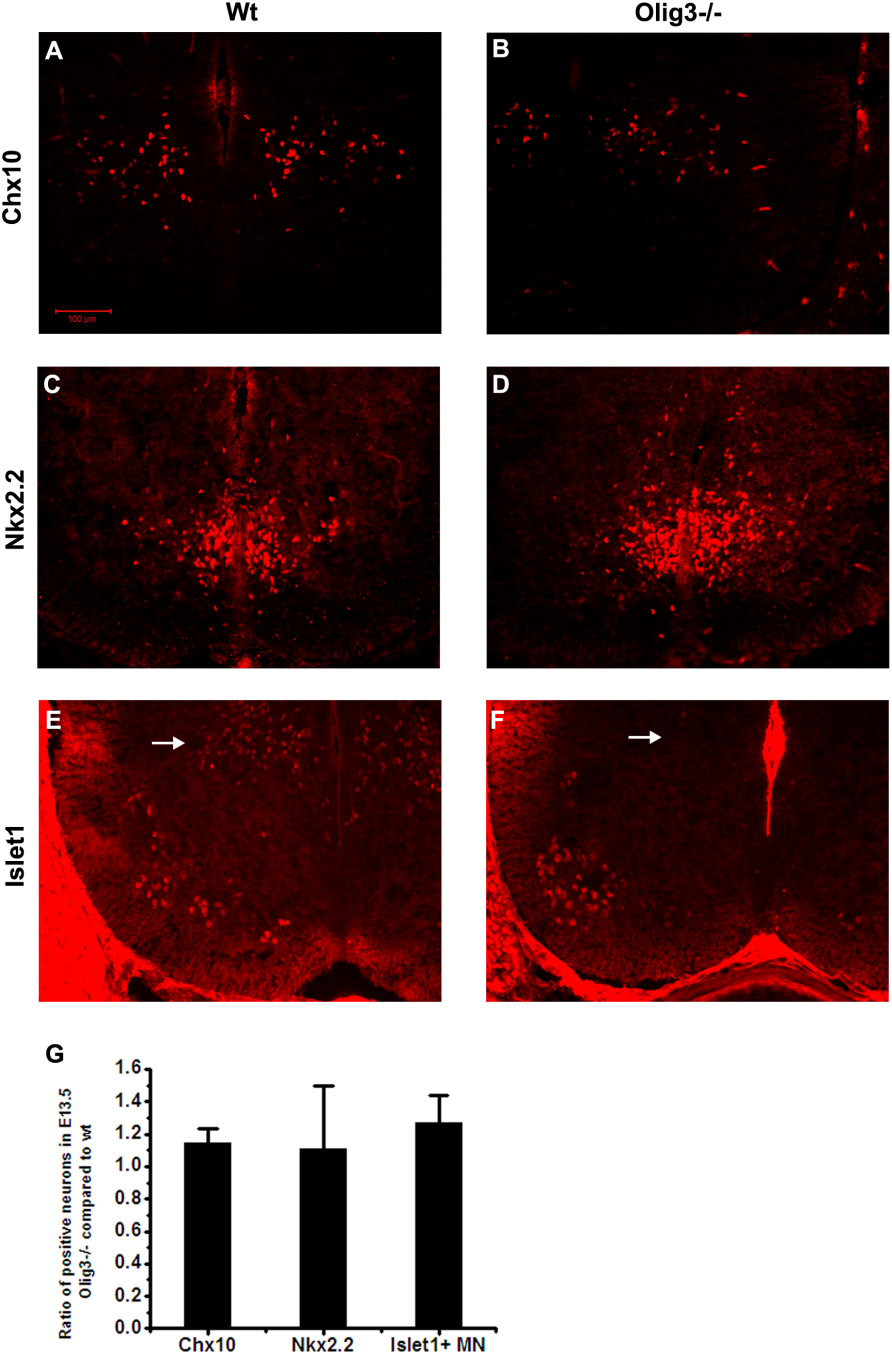
Loss of Olig3 function does not obviously affect the development of V2 and V3 interneurons and motor neurons. Spinal cord sections from E13.5 wild-type (A, C, E) and *Olig3*−*/*− (B, D, F) embryos were subjected to immunostaining with anti-Chx10 (A, B), anti-Nkx2.2 (C, D) and anti-Islet1 (E, F). The production and distribution of these interneurons was similar in wild-type and *Olig3*−/− spinal cords. Loss of Olig3 didn’t affect the generation of motor neurons, but inhibited the production of Islet1+dI3 interneurons. (G) The ratio of positive cells in *Olig3*−*/*− spinal cords compared to wild type. p>0.05. The dorsal part is up. Bars, 100 µm.

Double labeling experiments demonstrated that Olig3 was expressed in the postmitotic cells derived from p2 and p3 domains. Thus, we hypothesized that Olig3 may control the progenitor cells exiting from the cell cycle, then regulate the proliferation and differentiation of V2 and V3 interneurons. Since interneurons derived from the dI1, dI2, dI3, p0 and p3 domains of the spinal cord are involved in non-radial dorsal-ventral migration in the developing spinal cord [25], it is also possible that Olig3 may be directly involved in the promotion of interneuron migration. To explore these questions, Chx10 and Nkx2.2 expression was examined in E13.5 wild-type and *Olig3* mutant spinal cord. The results showed that the cell number and distribution of these interneurons were similar between wild-type and *Olig3*−/− spinal cords (Figure 8A–D). We also examined the development of motor neurons with anti-Islet1 antibody in *Olig3* mutants. The number of Islet1*+* motor neurons in the ventral spinal cord was counted, and there was no significant difference between the wild types and *Olig3* mutants (Figure 8E, F). However, Islet1+ dI3 interneurons were lost in *Olig3* mutants (Figure 8E, F), consistent with previous report that Olig3 is responsible for the correct specification of dI1–dI3 interneurons [12].

## Discussion

In the developing spinal cord, Olig3 is initially expressed in the dorsal-most progenitor cells of the spinal cord. One day later, Olig3 expression is also detected in the ventral marginal zone. In the ventral spinal cord, Olig3 is mainly expressed in post-mitotic differentiating neurons, but not in early neural progenitor cells. Birth-dating experiments indicated that the Olig3+ interneurons are born prior to E12.5, with the majority being produced at around E10.5 and E11.5 (Figure 4). While dorsal Olig3 expression is quickly down-regulated, Olig3 expression in the ventral spinal tissue persists until postnatal stages. Functional analysis revealed that dorsal Olig3 expression controls the early specification of dI1–3 interneurons, and loss of Olig3 activity reduces the number of dI1 interneurons and dramatically inhibits the generation of dI2 and dI3 neurons [12]. Meanwhile, ectopic dI4 neurons are generated, indicating the re-specification of dI2 and dI3 interneurons into dI4 neurons [11], [12]. The relatively late up-regulation of Olig3 in ventral neurons suggests that ventral Olig3 is not involved in the early specification of these neurons. Consistent with this idea, *Olig3* mutation does not affect the fate specification, proliferation or migration of these ventral interneurons.

In the ventral spinal cord, the majority of Olig3*+* ventral interneurons are derived from the p3 domain. In the development of V3 neurons, Olig3 seems to function downstream of Nkx2.2, as both the loss and gain of Nkx2.2 function dramatically affects the expression of Olig3 (Figure 6, 7). The strong and persistent expression of Olig3 in interneurons raises the possibility that ventral Olig3 may contribute to the establishment of various ventral interneurons, similar to other transcription factors (e.g. Evx1, Tlx3 or Lbx1) which are also up-regulated in postmitotic immature neurons. For example, Evx1 is expressed in post-mitotic V0 interneurons, and its expression can determine V0 interneuron identity [26]. In *Evx1* mutant embryos, V0 interneurons are transformed into V1 interneurons. In contrast, over-expression Evx1 can repress the expression of V1 interneuron marker En1 [26]. Both Tlx3 and Lbx1 are expressed in the post-mitotic neurons, but they play key roles in determining the cell fate of dI3–6 interneurons and late-born neuron dIL_A_ and dIL_B_ [27], [28], [29].

Surprisingly, disruption of Olig3 function has no apparent effect on the development of ventral interneurons, especially the V3 neurons. One possibility is the potential functional redundancy from other bHLH transcription factors (e.g. Sim1?) that are similarly expressed in these ventral interneurons. However, given that Olig3 is strongly expressed in V3 interneurons for a long period of time, it is also possible that Olig3 may be required for regulating the late differentiation events or morphogenesis of ventral V3 post-mitotic interneurons, such as the locomotor function and the axon projection. If this is the case, Olig3 plays different roles in regulating the development of dorsal and ventral spinal cord depending on its distinct temporal and spatial expression pattern. Since the conventional *Olig3* homozygous mouse die at birth due to the abnormal formation of respiratory center in the brainstem [11], further study on the conditional *Olig3* knockout mouse would be helpful to explore these possibilities.

## Supporting Information

Figure S1 **Genotyping of** ***Nkx2.2*** **and** ***Olig3*** **mutants.** Southern blot analysis of *Nkx2.2* (A) and *Olig3* (B) mutant. Genomic DNA was extracted from mouse tails, digested with restrictive enzymes and then hybridized with specific probes. Genomic DNA of *Nkx2.2* mutant was digested by ApaI, genomic DNA of *Olig3* mutant was digested by HindIII and SpeI. The sizes of wild-type and mutant alleles are indicated next to the DNA bands.
(TIF)

Figure S2 **Olig3 is expressed in V0, V2 and V3 interneurons at cE6.** cE6 chicken spinal cord sections were double-immunostained with anti-Olig3 (red), anti-Pax6 (green) and anti-Pax7 (green). Pax6 is expressed in the ventricular zone from pd1-pMN domain. Pax7 is expressed in the whole dorsal ventricular zone. Compared to Pax6 and Pax7 expression, Olig3 is expressed in the cells derived from p0, p2 and p3 domains of ventricular zone. The dorsal part is up. Bars, 100 µm.
(TIF)

Figure S3 **Olig3 is specifically expressed in neurons.** (A–C) Double staining showed that 92% Olig3+ cells co-express neuronal marker NeuN in cE9 spinal cord in the gray matter. (D–F) Olig3 is not expressed by Olig2+ oligodendroglia at cE12. The dorsal part is up. Bars, 100 µm.
(TIF)
