# Supplementary figures and images for: Olig3 Is Not Involved in the Ventral Patterning of Spinal Cord
Source: PLoS One. 2014 Oct 28;9(10):e111076. doi: 10.1371/journal.pone.0111076 (PMC4211884; doi:10.1371/journal.pone.0111076)

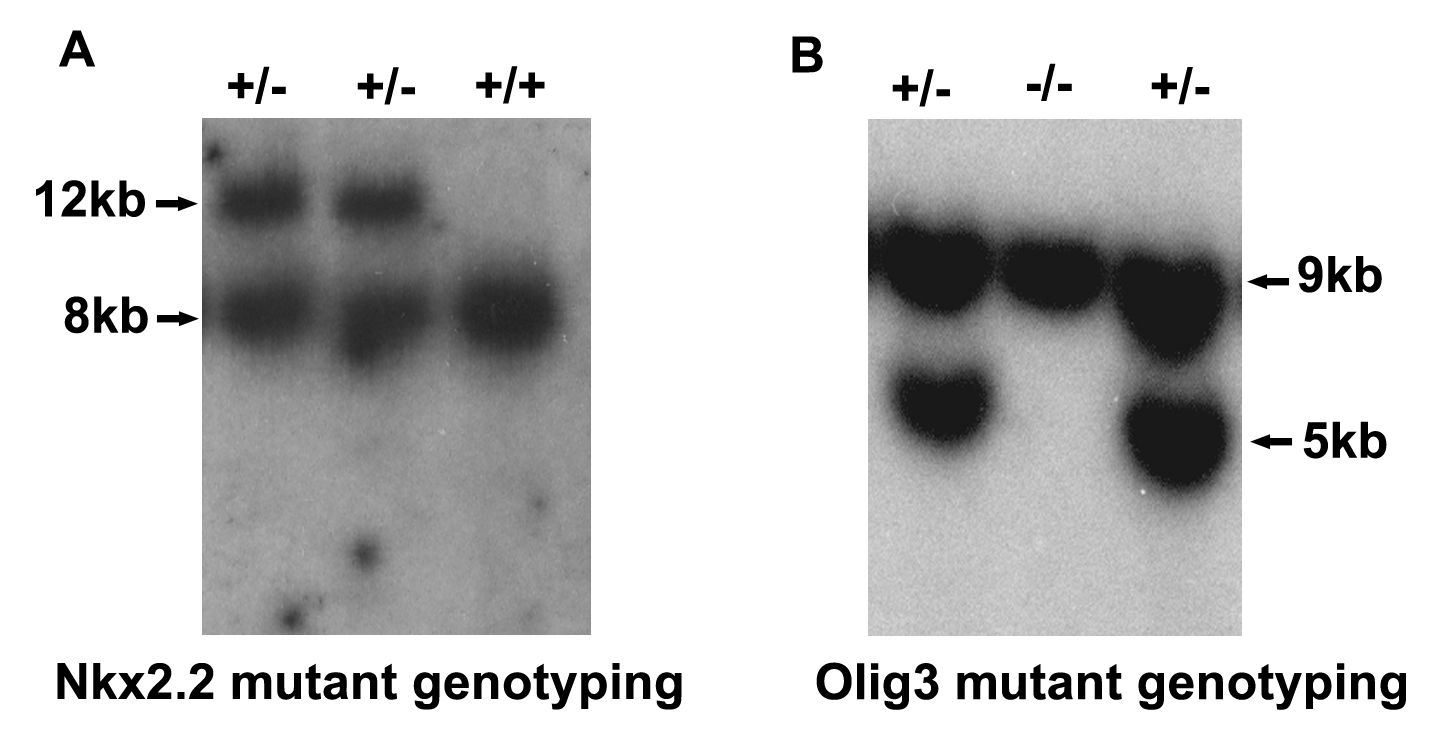

Supplement: Figure S1 — Genotyping of Nkx2.2 and Olig3 mutants. Southern blot analysis of Nkx2.2 (A) and Olig3 (B) mutant. Genomic DNA was extracted from mouse tails, digested with restrictive enzymes and then hybridized with specific probes. Genomic DNA of Nkx2.2 mutant was digested by ApaI, genomic DNA of Olig3 mutant was digested by HindIII and SpeI. The sizes of wild-type and mutant alleles are indicated next to the DNA bands. (TIF) [file pone.0111076.s001.tif]

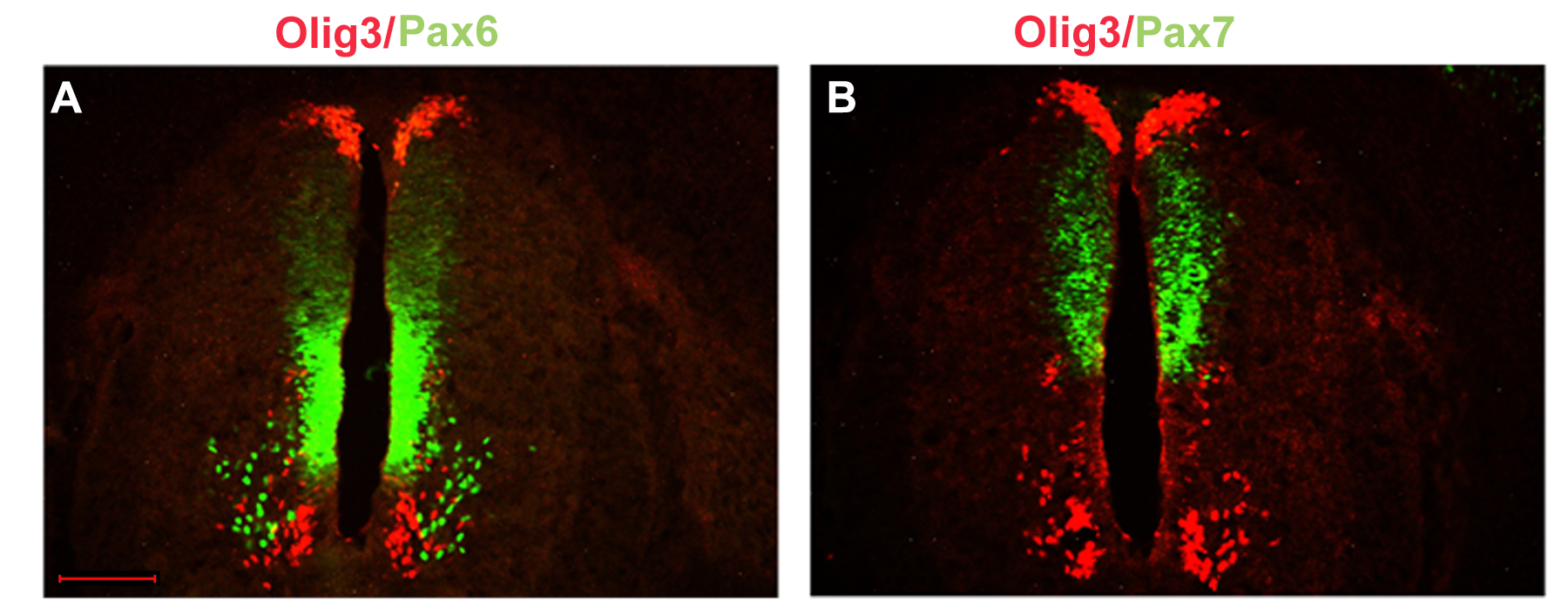

Supplement: Figure S2 — Olig3 is expressed in V0, V2 and V3 interneurons at cE6. cE6 chicken spinal cord sections were double-immunostained with anti-Olig3 (red), anti-Pax6 (green) and anti-Pax7 (green). Pax6 is expressed in the ventricular zone from pd1-pMN domain. Pax7 is expressed in the whole dorsal ventricular zone. Compared to Pax6 and Pax7 expression, Olig3 is expressed in the cells derived from p0, p2 and p3 domains of ventricular zone. The dorsal part is up. Bars, 100 µm. (TIF) [file pone.0111076.s002.tif]

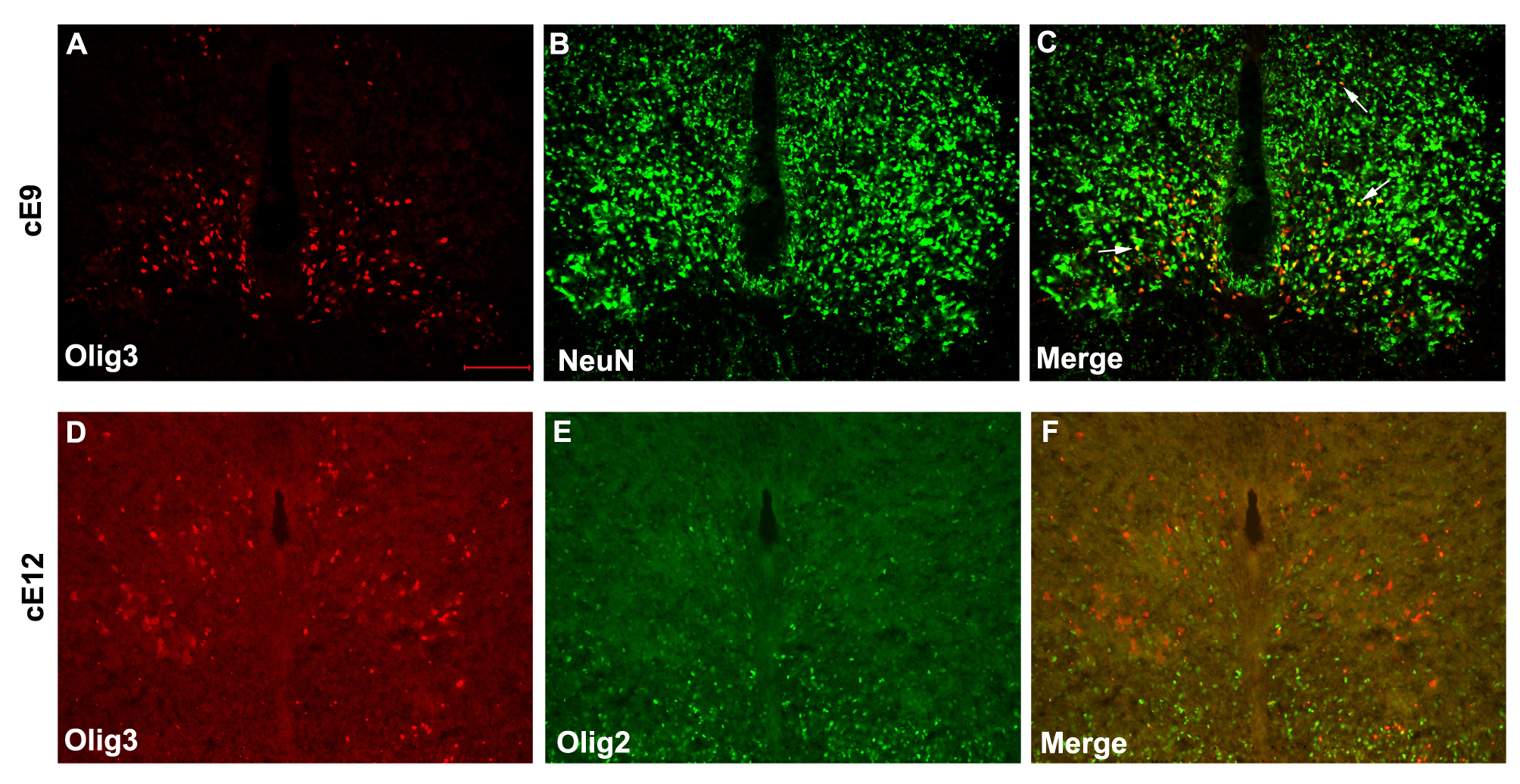

Supplement: Figure S3 — Olig3 is specifically expressed in neurons. (A–C) Double staining showed that 92% Olig3+ cells co-express neuronal marker NeuN in cE9 spinal cord in the gray matter. (D–F) Olig3 is not expressed by Olig2+ oligodendroglia at cE12. The dorsal part is up. Bars, 100 µm. (TIF) [file pone.0111076.s003.tif]
